# Circulating tumor cells in precision oncology: clinical applications in liquid biopsy and 3D organoid model

**DOI:** 10.1186/s12935-019-1067-8

**Published:** 2019-12-18

**Authors:** Chang Yang, Bai-Rong Xia, Wei-Lin Jin, Ge Lou

**Affiliations:** 1 Department of Gynecology Oncology, The Tumor Hospital, Harbin Medical University, Harbin, 150086 People’s Republic of China; 2 Institute of Nano Biomedicine and Engineering, Shanghai Engineering Center for Intelligent Diagnosis and Treatment Instrument, Department of Instrument Science and Engineering, Key Laboratory for Thin Film and Microfabrication Technology of Ministry of Education, School of Electronic Information and Electronic Engineering, Shanghai Jiao Tong University, Shanghai, 200240 People’s Republic of China; 3 National Center for Translational Medicine, Collaborative Innovational Center for System Biology, Shanghai Jiao Tong University, Shanghai, 200240 People’s Republic of China

**Keywords:** Circulating tumor cells, Liquid biopsy, Precision oncology, Tumor metastasis, 3D organoid model

## Abstract

Circulating tumor cells (CTCs) are a rare subset of cells found in the blood of patients with solid tumors, which function as a seed for metastases. Cancer cells metastasize through the bloodstream either as single migratory CTCs or as multicellular groupings—CTC clusters. The CTCs preserve primary tumor heterogeneity and mimic tumor properties, and may be considered as clinical biomarker, preclinical model, and therapeutic target. The potential clinical application of CTCs is being a component of liquid biopsy. CTCs are also good candidates for generating preclinical models, especially 3D organoid cultures, which could be applied in drug screening, disease modeling, genome editing, tumor immunity, and organoid biobanks. In this review, we summarize current knowledge on the value and promise of evolving CTC technologies and highlight cutting-edge research on CTCs in liquid biopsy, tumor metastasis, and organoid preclinical models. The study of CTCs offers broad pathways to develop new biomarkers for tumor patient diagnosis, prognosis, and response to therapy, as well as translational models accelerating oncologic drug development.

## Introduction

**Fig. 1:**
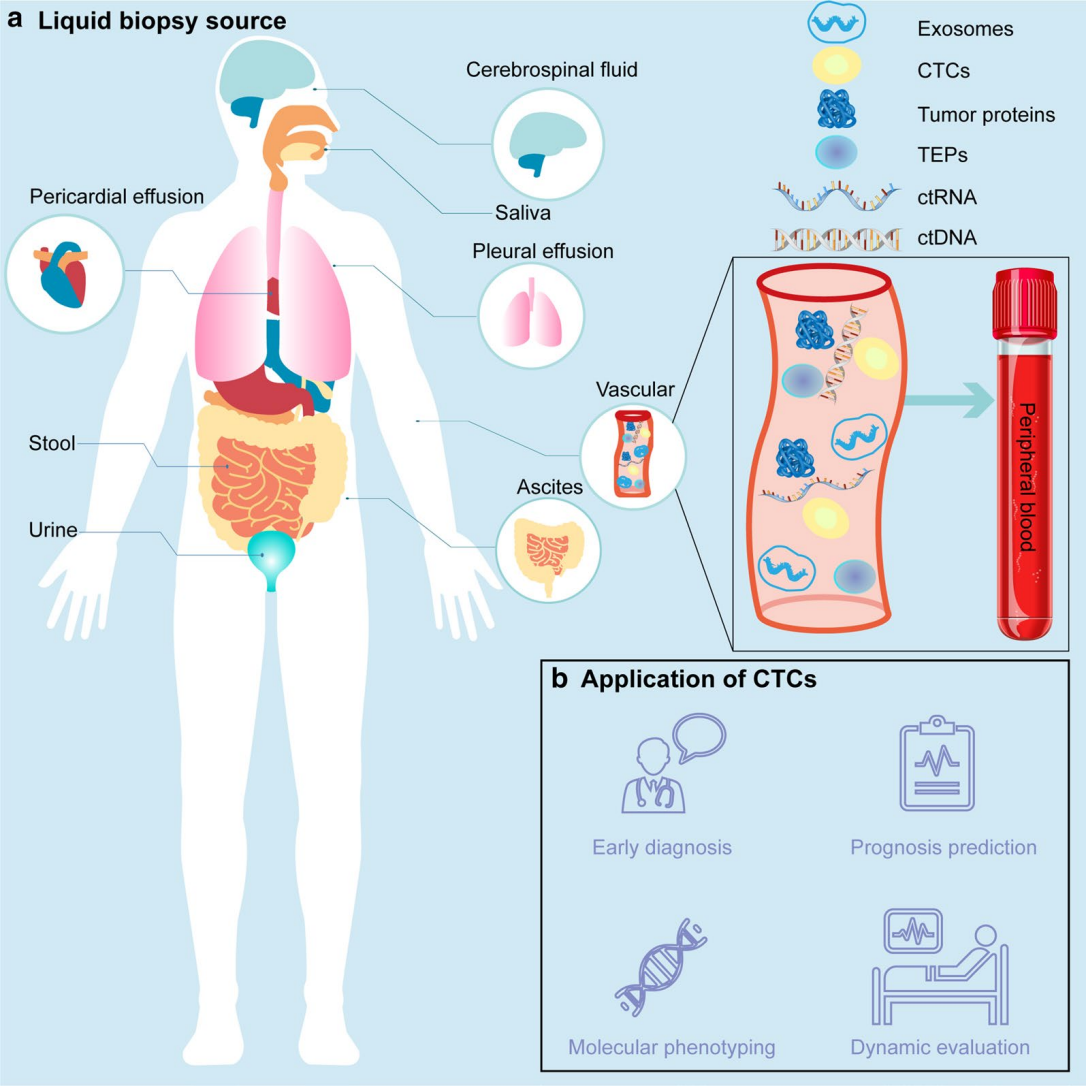
Basic introduction of the liquid biopsy approaches and applications of CTCs as liquid biopsy. **a** Liquid biopsy approaches involve peripheral blood, precardial effusion, stool, urine, ascites, pleural effusion, saliva and cerebrospinal fluid. Moreover, peripheral blood biopsy include isolation of circulating tumor cells(CTCs), circulating tumor DNA (ctDNA), circulating tumor RNA(ctRNA) and exosomes. **b** Applications of CTCs as liquid biopsy in early diagnosis, prognosis prediction, and disease monitoring, molecular phenotyping, therapy response evaluation

Although cancer incidence rate is stable in women and declining by approximately 2% per year in men, and cancer death rate in women and men declined annually by 1.4% and 1.8%, respectively, over the past decade [1], cancer remains the second leading cause of death globally and is responsible for an estimated 9.6 million deaths in 2018. Globally, approximately 1 in 6 deaths are due to cancer [2]. Limitations on the knowledge about cancer lead to high mortality. We routinely treat cancer patients with surgery, chemotherapy, and radiotherapy, ignoring inter- and intra-patient heterogeneity [3–6]. To address this issue, precision oncology is indispensable. Liquid biopsy [7], also known as fluid biopsy or fluid phase biopsy has potential in analyzing the genomic landscape of patients with cancer, supervising treatment responses, monitoring minimal residual disease, and managing non-invasive therapy resistance. Compared with traditional tissue biopsy, liquid biopsy is noninvasive and real-time. Blood samples are the most common materials for analysis, which contain cell-free DNA (cfDNA) [8]; cell-free tumor DNA (ctDNA) [9]; vesicles(such as exosomes [10]) tumor-educated blood platelets (TEPs) [11] and circulating tumor cells (CTCs) (Fig. 1a). Other body fluids such as cerebrospinal fluid (CSF) [12]; saliva [13]; pleural effusions [14]; urine [15] and stool [16] have shown captivity for diagnoses. CTCs play a vital role in precision oncology (Fig. 1b) due to its characteristics of non-invasion, real-time capability, and molecular heterogeneity.

As a seed for metastases, CTCs conserve tumor heterogeneity and mimic tumor properties, allowing them to be applied to therapeutic targets and clinical biomarkers for disease screening, dynamic monitoring, and prognosis prediction. Moreover, a CTC-derived 3D organoid model can be applied to screening tests of drug sensitivity [17] and analysis of multiplexed proteomic of CTCs [18]. Thus, although limitations exist, development of CTC isolation and culture are necessary for therapy, disease evolution, and real-time genomic characterization. In this review, we focus on the clinical applications of CTCs, especially in liquid biopsy and 3D organoid model.

## Technologies for CTC isolation and identification

**Fig. 2:**
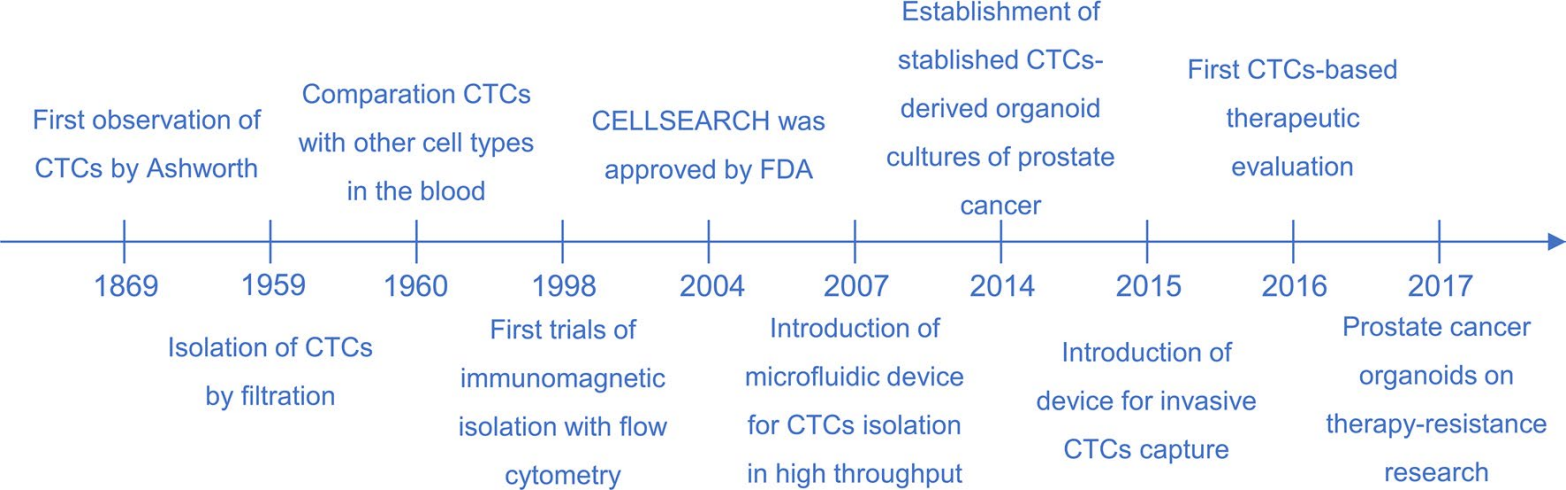
Milestones of CTCs development history

Circulating tumor cells (CTCs) with morphologic features similar to the primary solid tumor were initially discovered by Thomas Ashworth [19] through an autopsy of a cancer patient 150 years ago. A number of scientists have demonstrated that CTCs can be used as a predictor of clinical prognosis and treatment efficacy evaluation [20–23]. At first, scientists used the CellSearch system, which was the only device for CTC analysis approved by the United States Food and Drug Administration (FDA), to enrich and enumerate CTCs from peripheral blood. Finally, researchers discovered that the enumeration of CTCs is insufficient because variable phenotypes of CTCs in circulation have different potentials in tumor progress. Detailed developments [19, 24–30] in the history of CTCs are shown in Fig. 2.

Abnormal proliferation and metabolism of tumor cells, disorder and changes in the composition of cells, unnatural gene expression and modification, and synthesis and accumulation of polar particulate lead to changes in the physical and biological properties of CTCs. Scientists have developed technologies for enrichment, isolation, and identification of CTCs according to these physical and biological changes. The methods of technologies for CTC isolation from the review by Rubis [31], are referenced, but only the latest technologies are listed in Table 1. Methods to isolate CTCs developed rapidly with the emergence of the microfluidic chip system and nanotechnologies. Using engineered mouse models in cancer research, Hamza et al. [32] have solved the problem of having small total blood volume and rare CTCs using an optofluidic-based approach, eliminating confounding biases induced by inter-mouse heterogeneity. Antfolk et al. [33] have isolated breast cancer cells (MCF7) from peripheral blood with an efficiency of 91.8 ± 1.0% based on an integrated acoustophoresis-based rare-cell enrichment system combined with integrated concentration. Abdulla et al. [34] have introduced a cascaded microfluidic device that can separate 80.75% of human lung cancer cells (A549) and 73.75% of human breast cancer cells (MCF-7) from the human whole-blood system based on their physical properties within 20 min with a cell viability of 95% and 98%, respectively. Neves et al. [35] have constructed a glycan affinity-based microfluidic device for selective isolation of membrane protein *O*-glycan sialyl-Tn antigen (STn +), which are more sensitive than size-based microchips for CTC detection and are clinically relevant with metastasis in bladder and colorectal tumors. To conclude, technologies for enrichment, isolation, and identification of CTCs according to physical and biological changes both have limitations, such as low purity, low cell viability, and low intermediate throughput. It is urgent to integrate the best of these technologies to generate a new approach that yield high throughput and high purity. With the emergence of numerous technologies and platforms for isolating and further analyzing CTCs, physicians have realized the importance of CTCs as liquid biopsy and therapeutic target.

**Table 1:** CTCs isolation, enrichment, and identification technologies

Category	Strategy	Technology/device	Refs
Biological properties	Surface marker detection	Ferrofluids coated to EpCAM/CellSearch^®^	[81]
		Magnetic beads coated to EpCAM + MUC1/AdnaTest^®^	[111]
		EpCAM-coated wire for in vivo isolation/CellCollector™	[112, 113]
		CD45+ depletion	[114]
	Surface marker detection combined microfluid	Microposts or channels coated to EpCAM/CTC-Chip/HB-Chip	[25, 102]
		FICOLL and EpCAM-based microfluidic device/Isoflux^®^	[115]
		Glycan-affinity microfluidic devices	[35]
Physical properties	Size-based enrichment	ISET^®^	[116]
	Density-based	OncoQuick^®^	[117]
	Centrifugal force-based	cascaded microfluidic device	[34]
	Acoustophoresis-based	Acoustofluidic	[33]
	Nanorough polystyrene substrates adherence-based	Nanostructured polystyrene well plates	[118]
	Deformability-based	JETTATM	[119]
	Optofluidic-based	Optofluidic real-time cell sorter	[32]
	Dielectric-based	DEP	[120]
		DEP-LFFF	[121]
		DEP Array	[122]
Functional assays	Invasive capacity	VitaAssay™	[123]
	Protein release during culture	EPISPOT assay	[124]
	Telomerase expression	TelomeScan^®^	[125]

## Clinical application of CTCs

### Circulating tumor cells as a therapeutic target

As previously mentioned, CTCs are responsible for tumor metastasis. Furthermore, considering that most deaths induced by cancer are due to metastasis [36], a new cancer therapy that considers CTCs as a target is envisioned by scientists. Thus, the disruption of cancer cell dissemination would represent a powerful therapeutic strategy. However, owing to the lack of technical evaluation of the effects of CTC elimination in vivo, most studies assume that removing CTCs could radically prevent tumor metastasis. To address this condition, Kim et al. [37] have transplanted green fluorescent protein (GFP)-expressing CTCs into mice, applied photodynamic therapy to specifically clear GFP-expressing CTCs, appraised the therapeutic efficacy of CTC elimination, and finally demonstrated that elimination of CTCs could prevent metastasis and prolong the survival term of the tumor-bearing mice.

In recent years, Rana et al. [38] have established a selectin-based implantable shunt device based on the molecular mechanisms involved in CTC extravasation. The device is a microtube decorated with E-selectin molecules and tumor necrosis factor-related apoptosis-inducing ligand (TRAIL) in its surface to guide CTC rolling and the eventual tumor cell apoptosis. The application of TRAIL therapy into solid tumors is restrictive on account of TRAIL resistance. Phipps et al. [39] demonstrated that after separation from the extracellular matrix, TRAIL-resistant cancer cells became more sensitive. Furthermore, in the study by Mitchell [40], tumor cells showed enhanced sensitivity to TRAIL when exposed to fluid shear stress.

To conclude, CTCs, as a seed for metastasis, could be an effective therapeutic target toward limiting its recirculation in the blood, slowing its expansion to secondary lesions, and relieving overall tumor burden in cancer patients after the resection, radiation, or chemotherapy of a primary neoplasia. With an extensive study of the dynamics and mechanisms of CTC recirculation, a new therapeutic method tailored to oppose tumor seeding for advanced tumor patients is promising [31, 41].

### Circulating tumor cells in liquid biopsy

#### Prognosis prediction

The detection of CTCs should focus on identifying subpopulations of CTCs resulting in tumor metastasis because of the heterogeneous properties of CTCs [28, 42]. Moreover, learning the molecular and biological features of CTCs can guide clinical decision-making. Miyamoto et al. [43] have used microfluidic cell enrichment followed by digital quantitation of prostate-derived transcripts to predict metastasis and prognoses; they suggested that monitoring CTC-specific transcripts using this technology can guide clinical therapeutic selection in both malignant and regional prostate cancer. A secondary analysis of a randomized clinical trial has shown that the positive CTC assay of patients suffering from hormone receptor–positive breast cancer provided independent prognostic outcomes for late clinical recurrence, thereby indicating that CTCs may be used to predict late recurrence and guide therapy [44]. Importantly, a further step for the clinical application of CTCs in other carcinomas requires extensive validation.

#### Early diagnosis

Several lines of evidence suggest a crucial role of CTCs as a seed for metastases; thus, available data suggest CTCs as a biomarker for early diagnosis. Fan et al. [45] have designed a CTC panel to investigate the clinical value of circulating tumor cells for diagnosis in hepatitis B virus-related hepatocellular carcinoma. The panel showed prominent performance in early diagnosis and differential diagnosis from liver cirrhosis, chronic hepatitis B infection, and benign hepatic lesion. The area under the curve (AUC) of the CTC panel was 0.88 in training set and 0.93 in validation set. Recently, Zhou et al. [46] have demonstrated that folate receptor positive circulating tumor cells (FR+-CTCs), in combination with maximum tumor diameter (MTD), are reliable methods for determining whether small-sized solitary pulmonary nodules (SPNs) are invasive tumor or not. To conclude, CTCs may have promising beneficial effects in early diagnosis of tumor and may be relevant from the aspect of metastasis prevention.

#### Molecular phenotyping

PD-L1 antibody is an emerging anti-tumor regimen with less toxicity and long-term effects for a number of cancers such as non-small-cell lung cancer [47]. Mazel et al. [48] have demonstrated that the expression of PD-L1 highly increased on CTCs obtained from patients with hormone receptor-positive, HER2-negative breast cancer. CTC/PD-L1 analysis might be applied to patients with immune checkpoint blockade as immunoscores because PD-L1 expression categorizes different subsets of CTCs [49].

#### Dynamic evaluation

CTCs could be an independent indicator for evaluating tumor invasiveness and guiding clinical treatment because recurrence and metastases are hallmarks of cancer. Scher et al. [50] have used CTCs for therapy response evaluation in patients undergoing castration-resistant prostate cancer. The patients were randomly divided into abiraterone acetate plus prednisone and prednisone-alone groups. Moreover, the biomarkers were measured at baseline and at 4, 8, and 12 weeks. Results proved that CTC enumerations can be used for real-time therapy evaluation. Further trials are ongoing to validate the findings. Li et al. [51] have identified that the CTC levels after therapy may be used to evaluate therapeutic response and predict poor prognosis in advanced gastric cancer (AGC). They enumerated the newly diagnosed AGC patients’ CTCs as baseline and evaluated the first response after treatment by CellSearch in 136 patients. Moreover, they have chosen 15 appropriate patients and enumerated the CTCs during the entire treatment for a longitudinal study. In 2019, Balakrishnan et al. [52] have found that chemotherapy induced CTC cluster formation in blood samples indicate disease progression and shorter overall survival. To conclude, these studies may lead to a better understanding of the clinical application for CTCs on dynamic evaluation. To conclude, these studies may improve our understanding of the clinical application of CTCs in dynamic evaluation.

### CTC-derived 3D organoid model

**Fig. 3:**
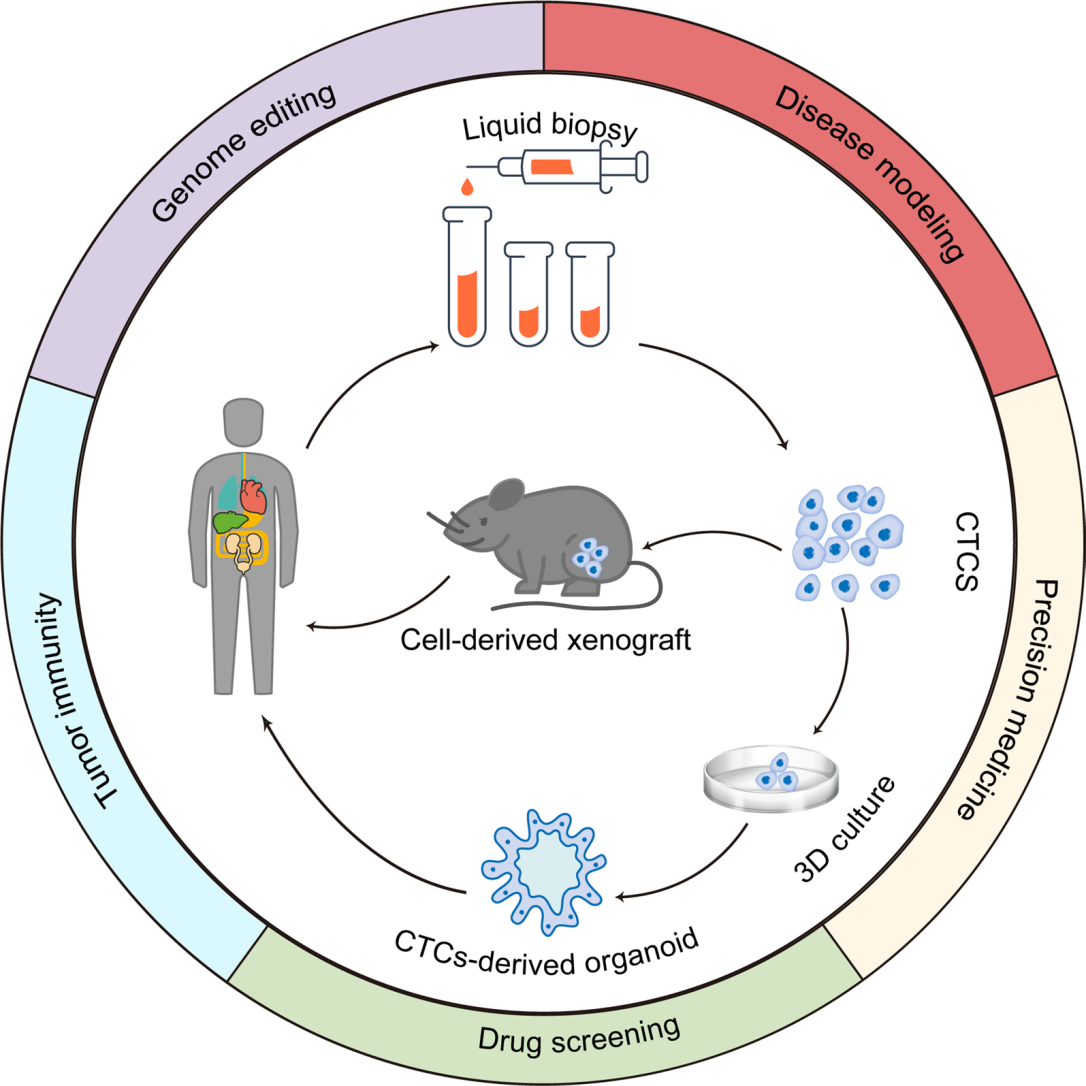
Basic introdction of CTCs-derived organoid in precision medcine

CTC-derived pre-clinical model consists of 2D cultures, spheroid generation, 3D organoid generation, and CTC-derived explant (CDX) model, which is an in vivo model compared with other mentioned models. The previous work of our group [53] have summarized that the 3D organoid model has advantages of stable morphology, gene expression and cell signaling, equal behavior and heterogeneity with cancer cells in the tumor mass, high-throughput for drug screening, low cost, and easy operation “in a dish” [54–58]. Moreover, organoids could mimic cancer hypoxia microenvironment. Thus, in this review, applications of organoid technologies in precision medicine are discussed in detail (Fig. 3).

#### Development of CTC-derived 3D organoid model

Organoids are miniscule models of tissues that grow in a 3D semisolid extracellular matrix medium with specific growth factor supplied [59, 60]. In the 1980s, Bissell et al. [61] developed 3D cultures and illustrated how extracellular matrix (ECM) affects gene expression in the breast. In 1990, her group further found that various ECMs play an important role in maintaining the morphology and function of murine mammary cells [62]. In 2007, Bissell et al. [63] proposed two procedures for 3D culture of normal and malignant human mammary cells. Formation of an organoid with single epithelial cells needs 7–10 days and the formed organoid can be separated into single cells to initiate a new organoid. This long-term organoid culture technology was first developed by Sato et al. [59], who cultured mouse small intestinal crypts in stable media condition for growth and finally established the mini-gut culture system. Subsequently, this technology was widely used in other organs including colon, lung, prostate, stomach, liver, pancreas, and breast for molecular research and drug screening [60, 64–68].

As CTCs are rare in blood, establish of CTCs-derived 3D organoid model are late. Zhang et al. [69] isolated CTCs from the peripheral blood of patients with breast cancer, cultured them in vitro, and finally developed CTCs lines. Moreover, they found that CTC lines maintained the characteristic of brain metastatic breast cancer (BMBC) were seriously invasive and metastatic. In 2014, Zhang et al. [70] have successfully designed a three-dimensional (3D) co-culture model for better isolating and culturing CTCs. After capturing CTCs from patients’ blood sample through a CTC-capture chip, they introduced fibroblasts and extracellular matrix (ECM) to the same chip to establish a co-culture environment, which could simulate a tumor microenvironment to support tumor development. Furthermore, it has been confirmed that CTCs in 3D co-culture model had matched mutation with the primary cancer, which could be applied into clinical application for evaluation of disease progress. Collectively, with the isolation and culture technology for CTCs evolution, CTCs-derived 3D organoids model will be widely used in clinical.

#### Potential application of CTC-derived 3D organoids

##### Disease modeling

Cancer progression is a multi-step accumulation process, such as angiogenesis, metastasis, and drug resistance, leading to difficulties in screening pathogenic gene events with specific stages in carcinogenesis. Organoids have a potential to model cancer and identify driver genes due to the convenient manipulation of retroviruses, inhibitors, and CRISPR/Cas9 approaches [58, 68, 71]. In 2015, Drost et al. demonstrated that organoids with triple mutations (APC^KO^, TP53^KO^, and KRAS^G12D^) showed slower growth than those with quadruple mutations (APC^KO^, TP53^KO^, KRASG^12D^, and SMAD4^KO^) within immuno-deficient mice injected with intestinal organoid model [58]. In 2017, Fumagalli et al., using the same model, proved that the subsequent mutation of oncogenes (APC^KO^, TP53^KO^, KRAS^G12D^, and SMAD4^KO^) promoted primary tumor growth, migration, and metastasis after orthotopic transplantation of organoids [71]. As CTCs could be obtained using a non-invasive method, it is easy to build a biobank for patients. Thus, in establishing biobanks of multiple organoid lines in different stages of same patients, CTC-derived organoids can help monitor metastatic progression.

##### Drug discovery

CTC-derived models that contain pathologies of patients are crucial for screening specific drugs. In 2014, Hodgkinson et al. [72] demonstrated that CTCs derived from patients with small-cell lung carcinoma mirrored the patient’s response to platinum, and etoposide treatment implied that CTC-derived explants could be applied in supervising the dynamic patterns of a tumor’s drug susceptibility and screening new therapeutic targets. In 2016, Boehnke et al. [73] successfully applied patient-derived colorectal cancer (CRC) organoids to high-throughput screening and drug discovery. In 2018, Sachs et al. [64] demonstrated that organoid lines generated from patient samples could be exploited to formulate a standard of care for different breast cancer subtypes. Therefore, various studies significantly showed the immense potential of organoid technology in revealing the molecular basis of drug response.

##### Precision medicine

A new concept in managing treatment programs, precision medicine considers individual differences of genes and environment (Table 2) [74]. In 2015, Van de Wetering et al. [75] reported that porcupine, a small molecule inhibitor of Wnt secretion, was viable only in a patient-derived organoid line with a mutation in the Wnt feedback regulator RNF43, implying the drug sensitivity in a subset of RNF43 mutation CRC patients. In 2017, Zhang et al. [76] proposed that CTC-derived organoid was useful in forecasting the therapeutic response to specific ALK inhibitors (ceritinib and crizotinib). In conclusion, CTC-derived organoids are available for drug screening based on the most recent genetic profiling, thereby settling the problem of drug resistance and invalid treatment.

**Table 2:** Summary of studies on CTCs in precision medicine

Category	Proposed functions	Representative genes	Refs
Oncogene validation	Epithelial mesenchymal transition (EMT)	TGFβ1, SNAIL1	[126, 127]
	Metastasis	FABP, CEACAM5	[128]
	Stem cell phenotype	CD24, CD44, CD133, ALDH1	[127]
	Cell proliferation	RRM1, MAPK14	[126]
Targeted therapy	Changing biomarker	HER2, EGFR	[129, 130]
	Signaling pathway	AKT1, AKT2, PIK3R1, PTEN	[131]
Drug screening	Biomarkers of therapeutic resistance	RAS, BRAF (colorectal cancer)	[132]
		AR (prostate cancer)	[30]
	Biomarkers of drug sensitivity	ER (endocrine therapy)	[133]
		ERCC1 (chemotherapy)	[134]
		PD1 (immune therapy)	[48]

##### Genome editing

CRISPR/Cas9, a technique that utilizes the mechanism of innate bacterial defense against bacteriophages, has been widely used in various fields of molecular biology since 2012. The indispensable roles of the combination of CRISPR and organoids focusing on the exploration of human tumorigenesis, heterogeneity, and metastasis have been summarized in the previous review [53].

##### Tumor immunity

Recently, cancer immunotherapies, such as CTLA-4 and PD-1/PD-L1, have sparked intense debate and research because of their substantial clinical benefits for advanced cancer patients. Dijkstra et al. [77] have established and confirmed a platform that culture autologous tumor organoids together with peripheral blood lymphocytes to evaluate and stimulate tumor-specific T cell responses to epithelial cancers. They have demonstrated that the value and novelty of this platform is to isolate tumor-reactive T cells and evaluate the therapeutic effect of T-cell-mediated attacks for the first time. Ultimately, with the improvement in success rate, this platform brings a bright prospect for patients with advanced cancer.

## CTCs and tumor metastases

**Fig. 4:**
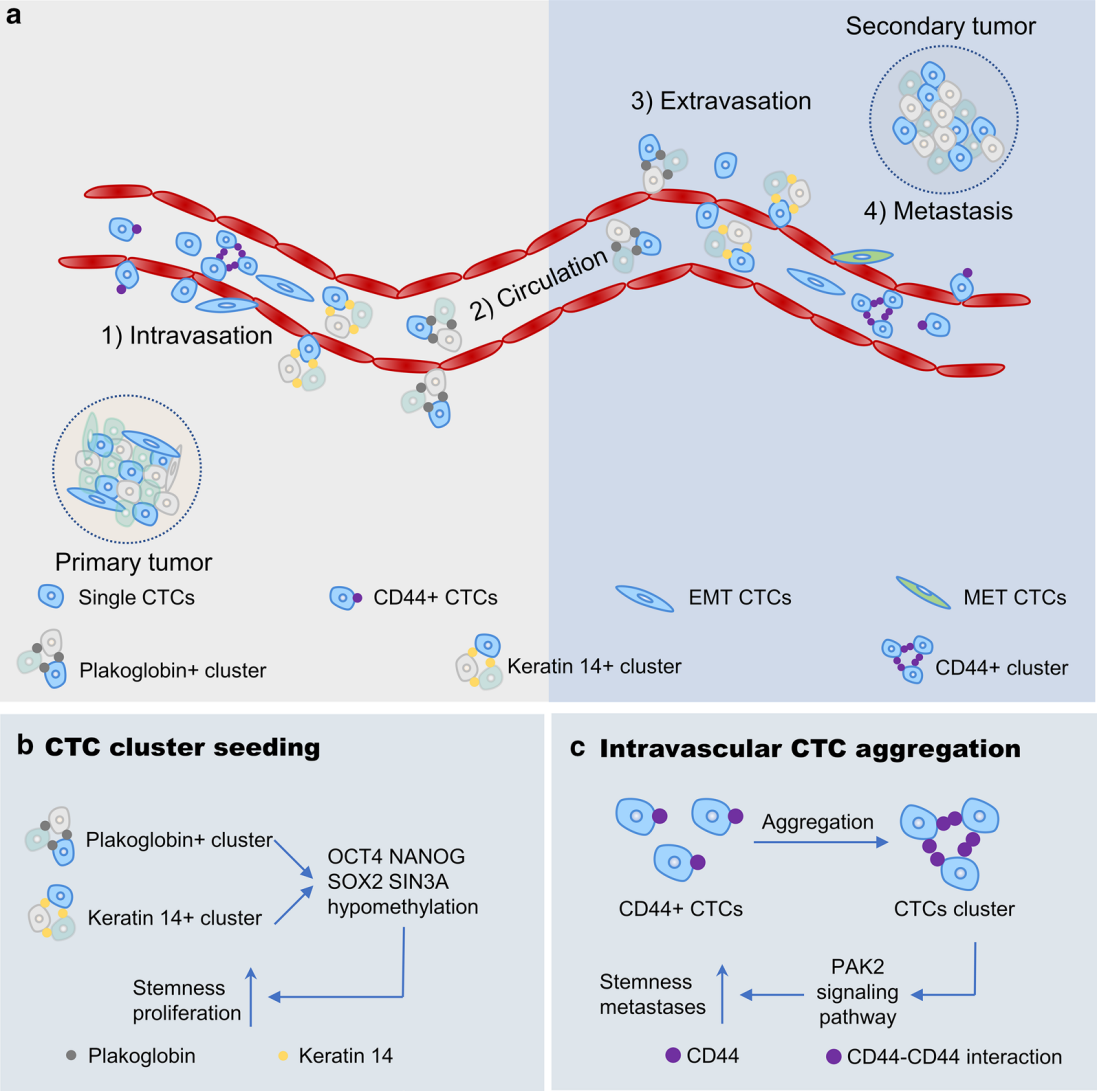
**a** Basic metastases process of CTCs and CTCs clusters. **b** CTC clusters seeding in blood. **c** Individual CTCs aggregation in blood

The motility and invasiveness of tumor cells initiate the onset of metastatic procedure [78], which consists of the steps: cancer cells separate from the primary tumor, seed in the blood circulation, sustain in circulation, extravasate into distant organs, and locate at secondary sites (Fig. 4a). Thus, finding CTCs in circulation indicates metastasis and poor prognoses in cancer patients [79–84]. However, not all CTCs are metastatic, since most of the CTCs in the circulation are degraded due to their half-life. Stott et al. have reported that the number of CTCs of 75% localized prostate cancer patients with preoperative CTCs declined precipitous after operation (< 24 h), which suggested a short half-life for CTCs in the blood circulation [85]. Here, two debate questions are discussed.

### Motility and mobility

Cancer metastasis has been correlated with genomics, transcriptomics, proteomics, and metabolomics [86–90]. However, the metastatic procedure of CTCs entering the blood circulation through an active course, passive process, or both, remains unclear [91]. In this review, we introduce the terms motility and mobility to describe the different procedures of tumor cell migration. Motile cancer cells move on their own because they have gained the abilities to seed in the extracellular matrix, undermine basement membranes, and penetrate and evade the vascular wall. The active invasive processes are based on the change of cell morphology, position, and surrounding tissue [92]. Mobile cancer cells are pushed by external forces dragging and pushing them out of place [93, 94]. Two probable mechanisms of passive dissemination of tumor cells are generalized: first, angiogenesis is one of the hallmarks of cancer by secretion of the vascular endothelial growth factor (VEGF), providing nutrients and oxygen for tumor growth. The junctions of neonatal vascular endothelial cells are loose and together with the pushing of tumor during growth result in tumor cell leakage [95]. Second, tumor cells may be passively following the route that was created by other tumor cells through proteolysis [96].

### Single cell and CTC cluster

Epithelial-to-mesenchymal transition (EMT) was debated as a way that initiates metastasis [97]. Alternative hypotheses have been proposed to illustrate the initiation of tumor metastasis from 1976 [98]. By using multiple technological platforms, Aceto and other researchers [99–103] identified 2–50 cancer cell clusters of CTCs from patients with metastatic epithelial cancers (Fig. 4b). Furthermore, in 2014, Aceto et al. [104] first demonstrated that CTC clusters generate from oligoclonal tumor cell groupings rather than from aggregating in the blood vessel. The number of CTC clusters are less than single CTCs, but metastasis is 23–50 times more potent than single CTCs. Aceto et al. [104] certified that plakoglobin-dependent intercellular adhesion promoted CTC clusters originating from connected multicellular groupings, and although less in number, these clusters largely contributed to the metastatic spread of cancer. In 2016, Cheung et al. [105] examined how polyclonal metastases form, and demonstrated that cancer cells transferred to distant organs as cohesive clusters composed of two molecularly distinct subpopulations with a variable ratio during metastasis. Moreover, the researchers identified that the mechanism for CTC cluster metastasis is related to keratin 14 + (K14+), which could regulate cell–cell adhesion, cell–matrix adhesion, and immune evasion. In 2019, Aceto and his colleagues [106] further found, based on their previous studies, that the binding sites of transcription factor for stemness and proliferation, including OCT4, NANOG, SOX2, and SIN3A are exactly hypomethylated in CTC clusters, and the biology of CTC clusters are analogous with that of embryonic stem cells. The researchers have confirmed this by profiling the difference of DNA methylation landscape between single CTCs and CTC clusters in breast cancer patients and mouse models on a genome-wide scale. Moreover, they identified Na +/K+ ATPase inhibitors from 2486 FDA-approved compounds to dissociate CTC clusters into single cells. Consequently, DNA methylation of transcription factor was remodeled at critical sites and the metastasis of tumor was suppressed. Finally, Aceto and his colleagues [106] evaluated the therapeutic effect of Ouabain after 3 weeks of treatment. Although the reduction of CTC clusters in mice blood with breast cancer led to a single increase in the number of circulating tumor cells, the total transfer in mice burden was reduced 80.7 times, 98.8% less than that in the control group, and prevented the formation of new metastases. Furthermore, Aceto pointed out that circulating tumor cell clusters are an important pathway for breast cancer metastasis, and the discovery of the first anti-circulating tumor cell cluster therapy may provide a powerful new tool to help treat millions of women currently living with this potentially fatal disease.

Interestingly, in contrast to Aceto and Cheung, Liu et al. [107] proposed by using intravital multiphoton microscopic imaging that CTC clusters were formed by a single tumor aggregation within the blood vessel rather than from communally migrating cell groups. This finding was confirmed by inoculating cancer cells into veins at different times. Additionally, the researchers revealed that CTC cluster aggregation was attributed to the interactions of homophilic CD44 and subsequent CD44-PAK2 interactions (Fig. 4c).

Further questions emerge from the three different studies. First, two mechanisms of the formation of CTC clusters exist simultaneously; thus, knowing which accounts for a major portion is essential to scientists in developing countermeasures against metastasis. Moreover, plakoglobin, keratin 14+, and CD44 are both involved in CTC aggregation, and whether they regulate tumor metastases in a separated or coordinated manner remains a question.

## Limitations and outlook

Although CTCs enable a non-invasive and dynamic analysis of cancer progress, limitations remain. First, CTCs are rare and extremely varied in different types of tumors. Similarly, various CTC detection methods have different boundaries of CTC enumeration to separate patients from the healthy group. To address this issue, the emerging microchip-based devices enable a high isolation efficiency and detection sensitivity of CTCs due to combination of microfluidic-based isolation techniques with nanomaterial-based detection systems into a single automatic platform.

Second, the heterogeneous nature of CTCs and recent research [28, 42] show that only certain subgroups of CTCs are capable of metastasis, and current information for detecting and identifying certain subpopulations are limited. Current techniques are already capable of downstream analysis of the released CTCs through culture expansion and single-cell analysis; thus, molecular phenotypes and biological features profiles might assist clinical diagnosis and treatments. In the future, CTC utility can be expanded to monitoring of immune responses of immune checkpoint or vaccination therapies, which can accelerate the translation of CTC research in the upcoming era of cancer immunotherapy.

Finally, CTC-derived 3D organoids are still characterized by limitations, such as lack of immune system, vascularization, and fibroblasts. In addition, these organoids cannot entirely recapitulate interactions at the tissue level in the human body and therefore cannot determine the rate-limiting organ toxicity of drugs [108]. However, microfluidic technology may be able to achieve co-culture of organoids and other cell types, such as immune cells, to imitate in vivo tumor microenvironment [109]. Further exploration is needed on whether CTC-derived organoids capture the complete heterogeneity of the carcinoma.

## Conclusion

CTCs have different physical and biological properties from peripheral blood cells, which can be used to develop new technologies for isolating, identifying, and relieving CTCs in high throughput. As techniques and methods evolve, translating fundamental research into clinical application can be expected. CTCs can be applied in liquid biopsy in early diagnosis, prognosis prediction, disease monitoring, molecular phenotyping, and therapy response evaluation. Moreover, metastasis begins with CTCs shedding from the primary tumor into the peripheral circulation. Recently, Klotz et al. [110] have cultured CTCs derived from patients with metastatic luminal breast cancers ex vivo. Intriguingly, a subset of them could adapt and grow in the brain. Therefore, therapies targeting CTCs can potentially reduce metastasis.

Moreover, the CTC-derived 3D organoid model plays a vital role in precision oncology because it can conserve tumor heterogeneity, imitate the cancer microenvironment, and maintain cancer oncogenesis and metastasis. This could gradually replace tissue biopsies which are painful and may be difficult to operate depending on the tumor location. To conclude, CTCs present a new dimension and horizon for clinical doctors in diagnosis, prognosis, prediction, treatment, disease mechanism, and drug development.

## Data Availability

Not applicable.

## Abbreviations

CTCs: circulating tumor cells
cfDNA: cell-free DNA
ctDNA: cell-free tumor DNA
TEPs: tumor-educated blood platelets
CSF: cerebrospinal fluid
FDA: Food and Drug Administration
STn: sialyl-Tn antigen
GFP: green fluorescent protein
TRAIL: tumor necrosis factor-related apoptosis-inducing ligand
AUC: area under the curve
FR+-CTCs: folate receptor positive circulating tumor cells
MTD: maximum tumor diameter
SPNs: solitary pulmonary nodules
AGC: advanced gastric cancer
CDX: CTC-derived explant
ECM: extracellular matrix
CRC: colorectal cancer
PD1: programmed cell death protein 1
PD-L1: programmed death-ligand 1
HER2: human epidermal growth factor receptor 2
CRISPR: clustered regularly interspaced short palindromic repeats
Cas9: CRISPR-associated protein 9
APCKO: adenomatous polyposis coli knock out
TP53KO: tumor suppressor p53 knockout
KRASG12D: the single-nucleotide mutation on codon-12 of exon-2 induces replacement of the GGT sequence (encoding for glycine) by the GAT sequence (aspartic acid-G12D-c35 G4A) of Kirsten rat sarcoma viral proto-oncogene
SMAD4KO: mothers against decapentaplegic homolog 4 knock out
RNF43: ring finger protein 43
ALK: anaplastic lymphoma kinase
CTLA-4: cytotoxic T-lymphocyte-associated protein 4
VEGF: vascular endothelial growth factor
EMT: epithelial-to-mesenchymal transition
K14: keratin 14
OCT4: octamer-binding transcription factor 4
NANOG: homeobox transcription factor Nanog
SOX2: SRY-Box 2
SIN3A: SIN3 transcription regulator family member A
CD44: hematopoietic cell E- and L-selectin ligand
PAK2: P21 activated kinase 2
TGFβ1: transforming growth factor beta 1
SNAIL1: zinc finger protein SNAI1
FABP: fatty acid-binding protein
CEACAM5: carcinoembryonic antigen
CD24: cluster of differentiation 24
CD133: prominin-1
ALDH1: aldehyde dehydrogenase 1 family, member A1
RRM1: ribonucleoside-diphosphate reductase large subunit
MAPK14: mitogen-activated protein kinase 14
EGFR: epidermal growth factor receptor
AKT1: AKT serine/threonine kinase 1
AKT2: AKT serine/threonine kinase 2
PIK3R1: phosphatidylinositol 3-kinase regulatory subunit alpha
PTEN: phosphatase and tensin homolog
RAS: Ras GTPase
BRAF: serine/threonine-protein kinase B-Raf
AR: androgen receptor
ER: oestrogen receptor
ERCC1: DNA excision repair protein ERCC-1
